# Low-Complexity Self-Interference Cancellation for Multiple Access Full Duplex Systems †

**DOI:** 10.3390/s22041485

**Published:** 2022-02-15

**Authors:** Shachar Shayovitz, Andrey Krestiantsev, Dan Raphaeli

**Affiliations:** School of Electrical Engineering, Tel Aviv University, Tel Aviv-Yafo 69978, Israel; andrey@mail.tau.ac.il (A.K.); danr@eng.tau.ac.il (D.R.)

**Keywords:** self-interference cancellation, full duplex, alternating minimization, recursive least squares, auto regressive process, multiple access

## Abstract

Self-interference occurs when there is electromagnetic coupling between the transmission and reception of the same node; thus, degrading the RX sensitivity to incoming signals. In this paper we present a low-complexity technique for self-interference cancellation in multiple carrier multiple access systems employing whole band direct to digital sampling. In this scenario, multiple users are simultaneously received and transmitted by the system at overlapping arbitrary bandwidths and powers. Traditional algorithms for self-interference mitigation based on recursive least squares (RLS) or least mean squares (LMS), fail to provide sufficient rejection, since the incoming signal is far from being spectrally flat, which is critical for their performance. The proposed algorithm mitigates the interference by modeling the incoming multiple user signal as an autoregressive (AR) process and jointly estimates the AR parameters and self-interference. The resulting algorithm can be implemented using a low-complexity architecture comprised of only two RLS modules. The novel algorithm further satisfies low latency constraints and is adaptive, supporting time varying channel conditions. We compare this to many self-interference cancellation algorithms, mostly adopted from the acoustic echo cancellation literature, and show significant performance gain.

## 1. Introduction

Future multiple access systems, supporting heterogeneous services and applications, such as virtual reality (VR), augmented reality, holographic telepresence, industry 4.0, and robotics, will have to accommodate multiple users in different resource blocks, such as time slots, frequency bands, spreading codes, and power levels. To accomplish this, these systems have to go to higher bandwidth efficiencies and higher connectivities compared to conventional multiple access schemes.

Full duplex communications, where both transmitter (TX) and receiver (RX) use the same frequency band at the same time, has the potential to improve the spectral efficiency of wireless communications and become a significant driver of the next generation of cellular communications. Full duplex communications can theoretically increase the spectral efficiency by a factor of two, and the flexibility of transmitting in any frequency at a given time may improve the scheduling performance. From the viewpoint of the base station, we denote the signals incoming from users as uplink (UL) and the system’s transmission as downlink (DL). These notations will be used interchangeably.

Many initial works on full duplex interference cancellations considered a single band operation [1,2,3]. In this simple case, the interference is present in all of the sampled bands and, therefore, resembles white noise, allowing simple algorithms, such as LMS or RLS, to be useful in self-interference cancellation. This is not the case in the carrier aggregation of contiguous and non-contiguous spectrum allocations. This technique is one of the main techniques used for increasing the capacity and flexibility of next generation wireless systems. Having analog filters on each of the tens of bands will not be practical, thus very wide band sampling will be used and simple solutions, such as RLS and LMS, will not work well.

Obviously, TX to RX interference occurs in the full duplex case, but it also appears in an additional case, where TX and RX reside in adjacent bands. Wideband sampling is used such that there is no analog filter at the transmitter to filter out the intermodulations caused by the power amplifier’s (PA) nonlinearity. This intermodulation noise limits the sensitivity of the victim RX band—and it is desired to measure it and cancel it. This is a similar model as the direct full duplex interference cancellation, so the same techniques hold in this case too.

**Figure 1:**
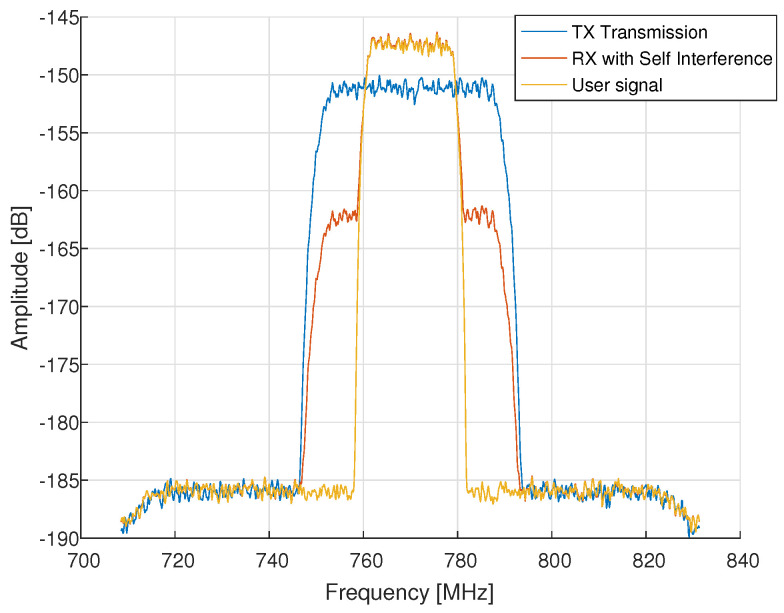
Exemplary spectrum of DL signal masking the UL signal.

An illustrative example can be seen in Figure 1, where a DL signal with 40 MHz bandwidth (BW) and a UL with 20 MHz BW are on the same center frequency. The DL leakage is added to the UL and can be seen more clearly in frequencies where the UL is not present. Consider an additional UL from another user transmitting a lower power signal at a frequency of 750 MHz (not shown). This user will suffer an increased noise level at the level of the DL leakage. Cancelling the DL signal while there is a strong UL signal in close frequency is the main difficulty treated in this paper.

There are several approaches to counter the effects of self-interference, which are generally composed of two steps. First, analog domain mitigation (antenna nulling or sharp analog filters) reduces the interference to a level that does not saturate the analog to digital converter (ADC). Next, digital signal processing algorithms counter the residual noise by estimating the leakage filter and then removing its interference on the UL. A comprehensive summary on the solutions and algorithms in both analog and digital domains can be found: [1,4,5,6,7,8,9]. For the rest of this paper, we will focus on the digital signal processing cancellation, assuming RX ADC is not saturated and the LNA is in the linear region.

Digital domain cancellation architectures can be generally divided to two categories: those using auxiliary path ADC, such as [7], and those which do not, such as: [2,3,10,11]. The signal after the digital to analog converter (DAC), in the TX RF path, passes through a PA and other active devices, which create nonlinear intermodulation (IMD) terms, which are hard to model accurately. The auxiliary ADC, sampling the signal as close as possible to the TX antenna, records an accurate replica of the TX signal, which can later be used for leakage filter estimation. Solutions that do not use an auxiliary ADC and, thus, save hardware costs, usually use some sort of polynomial approximation of the PA IMD. In [7], an auxiliary receiver measures the DL frequency response and a least squares (LS) estimation (not taking into account the UL spectrum) is performed to recover the leakage filter in frequency domain. Next, the filter is used to cancel out the self-interference signal by simply passing the DL through an inverse leakage filter. In [2], there is no auxiliary ADC path, and modeling of the IMD is proposed using second-order nonlinear terms. A training sequence is transmitted by the system when there is no RX reception and the self-interference filter is estimated using LS. This assumption is not useful in practice, since the users can transmit at any time, particularly in cellular communications. Furthermore, the channel may be changed during the payload, thus tracking is needed.

In the full duplex literature, estimation of the leakage filter is traditionally based on least squares (LS) techniques [9]. While these techniques are adequate for simple narrow band problems, they perform poorly for wide band systems in complex scenarios, which will be demonstrated in this paper. Least mean squares (LMS) is the most simple and low-complexity algorithm that can be used for a time-varying channel without any modification or loss. The main disadvantage of LMS is that it cannot converge if the input or the noise is highly correlated, which is the case in our application as explained above. We will later show in simulations that the LMS fails miserably. Another common family of algorithms is recursive least squares (RLS), which explicitly solves the LS problem in lower complexity, but even the RLS with its higher complexity will not help in many scenarios, since the interference is not white.

While the problem at hand was given less attention in the wireless full duplex community, we looked for candidate algorithms to compare to in other fields. Self-interference cancellation is analogous to a problem in acoustic research known as the acoustic echo cancellation. In this problem, the output of the loudspeaker (far-end signal) is fed back into the microphone, along with near-end signal (desired signal) and background noise. The far-end signal, can be viewed as the DL leakage signal while the near-end signal as the UL signal from users; the double-talk scenario is basically equivalent to the self-interference scenario. This problem has been extensively studied and there are a variety of techniques for echo cancellation that can be applied to our setting.

In [12,13,14], the variable step size normalized LMS (VSS-NLMS) algorithm is discussed, which is an extension of the LMS algorithm. This algorithm is designed to overcome the difficulty of gradient noise amplification by normalizing the step-size parameter by the power of the input signal. In basic NLMS algorithms, the step-size parameter is constant and the choice of the variable step-size parameter reflects the trade-off between speed of convergence, on the one hand, and achieving a small misadjustment, on the other hand. In [15], the affine projection algorithm (APA) is presented. This algorithm is a generalization of the NLMS algorithm, which uses N (called projection order) vectors of the input signal instead of a single vector as the NLMS algorithm. Under this interpretation, NLMS can be viewed as a one-dimensional affine projection algorithm. As the projection dimension increases, so does the convergence speed of the algorithm and, unfortunately, the algorithm’s complexity.

The family of the multi-delay filtering (MDF) [16] algorithm was proposed to mitigate the self-interference problem by partitioning the adaptive filter length *L* into shorter length-*N* sub-filters, such that the delay was reduced by a factor of K=L/N. Improved proportionate MDF (IPMDF) [16] was proposed for networks where the impulse response was sparse. In [17,18], the extended MDF (eMDF) considered the correlation between blocks of MDF.

Subband filtering is another approach used to tackle the fact that the UL is not spectrally flat. The transmitted and received signals are divided into narrow frequency bands by filter banks. Since the UL is assumed to be relatively flat at each band, LMS can be applied to each one separately. One of the disadvantages of subband filtering is the additional delay caused by the filtering performed by the analysis and the synthesis filter banks. Another disadvantage of subband filtering is that the stopbands of different subbands can alias and leak into other subbands; therefore, filters should be sharp, which results in many taps and, thus, increases complexity, power, and delay. Another important issue with subband filtering is the fact that downsampling and low pass filtering to subbands can cause the adaptive filters in the subbands to become non-causal, and inducing delays into the echo path may become necessary, which further increases the delay caused by the subband filtering [19]. Additionally, when time varying leakage has to be tracked, the convergence will be extremely slow; thus, channel variations cannot be tracked.

Kalman adaptive filtering [20,21,22] makes the assumption that a time-varying echo path changes slowly and can be modeled by a first order Markov model. However, this approach is highly affected by the covariance matrices of process noise and measurement noise, and improper choices of these statistics may significantly degrade the Kalman filter performance. Estimation of these covariance matrices adds significant complexity to the algorithm.

Most of the above algorithms for digital domain cancellation perform fairly well when the UL and DL are spectrally white. In fact, performance of LS, RLS, and LMS will be as good as maximum likelihood (ML) only when the UL is either spectrally white or its power is significantly lower than the self-interference. However, in practical applications, in particular multiple access communications, multiple carriers at arbitrary bandwidths and power levels coexist at the UL; thus, its spectrum is non-white and its power might be comparable to the leakage.

In this paper, we propose a novel algorithm for interference cancellation, which is robust to the spectrum shape of the DL and UL and provides high rejection in highly non-flat scenarios. The algorithm, first proposed in [23], is based on the observation that the UL signal can be approximated as an autoregressive (AR) process and a self-interference cancellation algorithm utilizing the special characteristics of the AR process was devised. The algorithm performs an approximate joint maximum likelihood estimation of the leakage and AR filters using alternating minimization. In subsequent sections, we will show simulations of scenarios where state-of-the-art algorithms fail to provide sufficient interference rejection, while our novel algorithm provides dramatically better interference rejection. The main advantage of our approach is in scenarios where the UL contains a strong narrow band user along with other weak users, while interference from DL affects the weak user. In such scenarios, the leakage filter is poorly estimated by traditional methods and the weak users are not serviced. Our algorithm is able to remove the self-interference such that these users can be serviced, which results in an increased service range of the system.

The resulting algorithm can be implemented online using a low-complexity architecture composed of only two RLS modules, providing real time tracking for variations in the model of the UL and the leakage channel. The RLS blocks can be implemented using low-complexity methods, such as DCD [24], providing overall practical, robust, low-complexity system implementation and latency.

This paper adds to the work presented in [23], by extending it to a more practical system, having a sampler that samples the signal close to the transmitter and adding another receiver chain. Moreover, no sufficient evaluation of the algorithm was done in [23]. In this paper, we perform a comprehensive complexity and performance comparison between the proposed algorithm and many state-of-the-art algorithms. The analysis presented demonstrates the superior rejection performance of our algorithm compared to state-of-the-art algorithms, while having a relatively low computational complexity. In addition, the rejection performance of the algorithm for time varying channels is simulated and it is shown that the algorithm performs well for typical time-varying channels using Jakes’ channel model [25]. Finally, a proof for convergence of the algorithm is provided.

## 2. System Model

**Figure 2:**
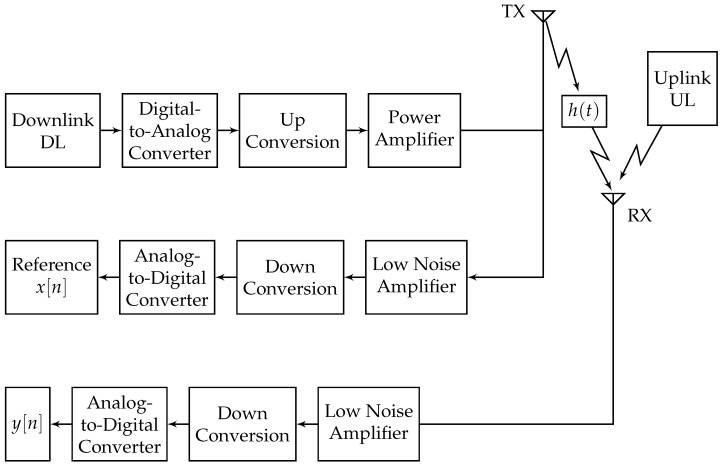
System architecture.

In this section, the system model for our setting is presented and a base band model is derived. A block diagram of the communication system is presented in Figure 2. We note that there is a dedicated reference channel that samples the DL signal as close to the TX antenna as possible for use as a reference. Sampling the TX signal requires a coupler, which attenuates the signal toward the reference ADC. By sampling the TX signal after the PA, the intermodulations are taken as part of an effective transmitted signal, which is used as the reference. Moreover, as described in Figure 2, two identical RX analog processing chains are used for the RX and reference signals. The same LO is used both in the reference branch and RX branch for down-conversion and, thus, phase noise is cancelled out. Adding another RX channel is justified only when there is a formidable nonlinear part in the PA, otherwise the reference can be the digital TX signal itself. In addition, LNA linearity impairments on the RX signal are neglected since it is assumed that there is some analog cancellation before LNA, such as antenna nulling, which provides sufficient initial self-interference rejection (35–40 dB initial rejection of self leakage).

Let y[n] be the discrete time domain received signal after down-conversion and sampling, which is composed of the DL self-interference and UL signals. Let x[n] be the discrete time domain reference signal.

Since the proposed algorithm deals with the digital samples, we can model the ADC output vector $\underline{y}$ of *N* samples or y[n] as,

(1) $$\underline{y}=X\underline{h}+\underline{s}$$

where *s* is the UL, modeled as a size *N* circularly symmetric complex normal random vector, *h* is the self-interference filter of length *M* and *X* is an N×M tall Toeplitz matrix (N≫M) with $X_{ij}=x\left[i+j\right]$ for 0≤i<N and 0≤j<M. The matrix multiplication $X\underline{h}$, is the equivalent of convolving the digital DL with an FIR filter: $\underline{h}$ (neglecting boundary effects). The FIR filter is a filtered and sampled approximation of the analog leakage filter.

Moreover, we assume a Bayesian setting where the self-interference filter is a circularly symmetric complex normal random vector $\underline{h}∼\mathrm{CN}\left(0,\;\sigma_{h}^{2}I\right)$. This is only really needed as part of the regularization for the leakage filter estimation at low signal-to-noise ratio (SNR) scenarios.

## 3. Proposed Solution

As discussed in the introduction, most digital domain self-interference mitigation algorithms attempt to estimate the leakage filter using some variants of LS. Without prior information on the UL signals, the common working assumption is to model the UL as an AWGN process. However, in the multiple access scenario, the UL signal is composed of multiple carriers/users with different bandwidths and power levels. For example, several LTE and CDMA carriers from multiple users. Therefore, the UL is clearly not spectrally white and, thus, RLS and LMS will have a significant performance loss compared to ML with prior information on the UL spectrum.

The first step in our algorithm derivation is finding a statistical model to approximate the UL signal. The autoregressive moving average (ARMA) model defines a dense set in the class of all continuous PSDs according to Section 3.2 in [26]. These processes are modeled as the output of a stable LTI system with zero mean white Gaussian input, where the frequency response of the system can be written as a division of two polynomials. Therefore, the second order statistics of an ARMA process can approximate most well-behaved WSS processes. However, ARMA processes are hard to estimate and it is preferred to work with AR processes instead. Fortunately, causal and invertible ARMA processes can be written as an AR process of infinite order [27]. Therefore, we suggest approximating the UL signal $\underline{s}$, as a complex valued autoregressive process of order *p*, which is fine-tuned by the users to achieve maximum performance. Obviously, using a finite model order *p* is only an approximation of the true UL statistical model, and as *p* increases to infinity, the model may approach the true spectrum of the UL, assuming the UL is WSS.

### 3.1. Stochastic Modeling of the UL Signal

The AR model approximating the UL signal will be written as

(2) $$s\left[n\right]=\sum_{k=1}^{p}g_{k}s\left[n-k\right]+u\left[n\right]$$

where $\underline{g}=\left[g_{1},g_{2},\ldots ,g_{p}\right]$ is an unknown vector of size *p*, u[n] is a circularly-symmetric complex normal i.i.d process with zero mean and variance $\sigma_{u}^{2}$. The choice of *p* determines the approximation accuracy, and it affects the model’s frequency selectivity.

Equivalently, (2) can be written in matrix form,

(3) $$\underline{u}=W\underline{s}$$

where *W* is a square Toeplitz whitening matrix with dimension *N*, which is the size of vectors $\underline{u}$ and $\underline{s}$

(4) $$\begin{array}{cc} \; & W=\left(\begin{array}{ccccccccc} 1 & -g_{1} & -g_{2} & \ldots & -g_{p} & 0 & 0 & \ldots & 0\\ 0 & 1 & -g_{1} & -g_{2} & \ldots & -g_{p} & 0 & \ldots & 0\\ \ldots & \ldots & \ldots & \ldots & \ldots & \ldots & \ldots & \ldots & \ldots \\ 0 & 0 & 0 & 0 & \ldots & 0 & 0 & 1 & -g_{1}\\ 0 & 0 & 0 & 0 & \ldots & 0 & 0 & 0 & 1 \end{array}\right) \end{array}$$

We notice that due to (3) and the fact that *W* is invertible, the co-variance matrix Σ of the random vector $\underline{s}$ can be written as,

(5) $$\Sigma =E\left({\left(W^{-1}\right)}^{†}\underline{u}{\underline{u}}^{†}W^{-1}\right)$$

which is,

(6) $$\Sigma ={\left(W^{-1}\right)}^{†}E\left(\underline{u}{\underline{u}}^{†}\right)W^{-1}$$

Since $\underline{u}$ is an i.i.d vector, the inverse is

(7) $$\Sigma^{-1}=\frac{W^{†}W}{\sigma_{u}^{2}}$$

### 3.2. Minimum Mean Square Error Estimation of the UL Signal

The second part of the algorithm derivation is to recover the UL signal, and we choose to use the minimum mean square error (MMSE) criterion. Therefore, the optimal estimator for $\underline{s}$ under the block signal model in (1) is

(8) $${\hat{\underline{s}}}_{MMSE}=E\left(\underline{s}|\underline{y}\right)$$

where,

(9) $$\begin{array}{cc} {\hat{\underline{s}}}_{MMSE} & =E\left(\underline{y}-X\underline{h}|\underline{y}\right)\\ & =\underline{y}-XE\left(\underline{h}|\underline{y}\right)\\ & =\underline{y}-X{\hat{\underline{h}}}_{MMSE} \end{array}$$

Since $\underline{h}$ is assumed to be a random Gaussian vector, then the MMSE solution is the same as the maximum likelihood (ML). The ML solution for the leakage filter finds the vector $\underline{h}$, which maximizes the log likelihood function:

(10) $$\begin{array}{cc} \log\left(p(\underline{y}|\underline{h};\Sigma )\right) & \propto -\log(\det\Sigma )\\ & -{\left(\underline{y}-X\underline{h}\right)}^{†}\Sigma^{-1}\left(\underline{y}-X\underline{h}\right) \end{array}$$

where Σ is the covariance matrix of the vector $\underline{s}$ and ${\left(\right)}^{†}$ is the matrix conjugate transpose operator.

If Σ was known a-priori, then maximization of (10) would reduce to a closed form solution (weighted least squares (WLS)). Moreover, if $\underline{s}$ was an i.i.d vector, then the LS and RLS solutions would yield the same performance as ML.

However, *s* has unknown statistics, since it is the combination of all the active users in a given cell sector. All users are transmitting in different bandwidths, center frequencies, and power levels, in various formats, such as LTE, GSM, and WCDMA.

We propose using a generalized likelihood ratio (GLRT) approach for solving the ML problem. We find the vector $\underline{g}$, which maximizes (10), and use it to compute the posterior. Equivalently, we can look at this approach as jointly maximizing (10),

(11) $$\hat{\underline{h}},\hat{\underline{g}}=\arg\max_{\underline{h},\underline{g}}p\left(\underline{y}|\underline{h};W\right)$$

### 3.3. Alternating Minimization Algorithm

In this section, an algorithm that approximately solves (11) is proposed. Since the optimization is also done on the matrix *W*, then the problem does not have a simple closed form solution and a unique algorithm is developed. We use alternating minimization [28] of the likelihood function and converge on a joint solution for both filters.

Note that since $\det A^{-1}=\frac{1}{\det A}$, (10) becomes,

(12) $$\begin{array}{cc} \log\left(p(\underline{y}|\underline{h};\Sigma )\right) & \propto \log(\det W^{†}W)\\ & -{\left(\underline{y}-X\underline{h}\right)}^{†}\frac{W^{†}W}{\sigma_{u}^{2}}\left(\underline{y}-X\underline{h}\right) \end{array}$$

Since *W* is an upper triangular matrix with all ones across its main diagonal (assuming large enough vectors, thus neglecting boundary effects), then detW=1. Moreover, $\det W^{†}W=\det W^{†}\det W$; thus $\det W^{†}W=1$

(13) $$\begin{array}{c} \hat{\underline{h}},\hat{\underline{g}}=\arg\min_{\underline{h},\underline{g}}{\left(\underline{y}-X\underline{h}\right)}^{†}W^{†}W\left(\underline{y}-X\underline{h}\right) \end{array}$$

In the following sections, the details of the alternating minimization algorithm will be discussed. In particular, the alternating minimization algorithm can be converted into two alternating least squares problems, based on the specific structure of *W*.

The flow diagram of the algorithm is detailed in Algorithm 1, which works in batch on the received signal, $\underline{y}$. In the start, ${\underline{h}}^{k=0}$ and ${\underline{g}}^{k=0}$ are set to some initial values, where *k* is the iteration index for the alternating minimization. Then ${\underline{h}}^{k}$ is fixed and a minimization for ${\underline{g}}^{k+1}$ is performed. Then ${\underline{g}}^{k+1}$ is fixed to the new value and ${\underline{h}}^{k+1}$ is optimized. This procedure is repeated until the difference between the estimated filters in subsequent iterations is smaller than a predefined value. Note that the algorithm uses diagonal loading factors $\lambda_{h}$ and $\lambda_{g}$. These parameters are commonly used for regularization in LS problems. The regularization introduces the terms ${∥{\underline{h}}^{k}∥}^{2}$ and ${∥{\underline{g}}^{k}∥}^{2}$ (leakage and whitening) into the LS optimization, thus, improving the robustness of the LS estimation to low SNR situations.
**Algorithm 1:** Batch alternating minimization algorithm.
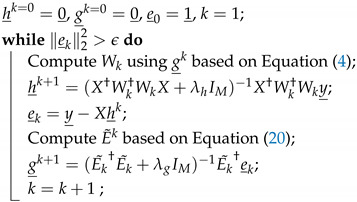


### 3.4. Minimization over the Self-Interference Filter

We used the previous estimation of the AR filter taps, ${\underline{g}}^{k}$, which defines the matrix $W_{k}$ and minimizes (13) over the vector $\underline{h}$.

(14) $${\underline{h}}^{k+1}={\left(X^{†}W_{k}^{†}W_{k}X+\lambda_{h}I_{M}\right)}^{-1}X^{†}W_{k}^{†}W_{k}\underline{y}$$

where $I_{M}$ is an *M* by *M* identity matrix and $\lambda_{h}$ is a regularization factor added to increase robustness to the estimation.

Note that (14) is the WLS solution to the ML problem, when the matrix, $W_{k}$, is known. This result has the following interpretation: passing the DL and UL signals through a whitening filter and performing LS estimation of the self-interference filter. In the next step, we will use this filter estimation in order to estimate the whitening filter; this process will alternate until convergence.

### 3.5. Minimization over the Whitening Filter

In the second step of each iteration, we use the previous estimation of the self-interference filter, ${\underline{h}}^{k}$ and minimize (13) over the vector $\underline{g}$.

We define the following residual vector:

(15) $${\underline{e}}_{k}=\underline{y}-X{\underline{h}}^{k}$$

Plugging (15) into (13)

(16) $$\begin{array}{c} {\underline{g}}^{k+1}=\arg\min_{\underline{g}}{∥W{\underline{e}}_{k}∥}^{2} \end{array}$$

Since *W* is a Toeplitz matrix dependent on $\underline{g}$ and the matrix multiplication in (16) is equivalent to a convolution between $\left[1,-\underline{g}\right]$ and ${\underline{e}}_{k}$, we can rewrite (16) as,

(17) $$\begin{array}{c} {\underline{g}}^{k+1}=\arg\min_{\underline{g}}{∥E_{k}\left(\begin{array}{c} 1\\ -\underline{g} \end{array}\right)∥}^{2} \end{array}$$

where $E_{k}$ is a Toeplitz matrix defined as,

(18) $$\begin{array}{cc} \; & E_{k}=\left(\begin{array}{cccc} e_{k}\left(N\right) & e_{k}\left(N-1\right) & \ldots & e_{k}\left(N-p\right)\\ e_{k}\left(N-1\right) & e_{k}\left(N-2\right) & \ldots & e_{k}\left(N-\left(p+1\right)\right)\\ \ldots & \ldots & \ldots & \ldots \end{array}\right) \end{array}$$

where $e_{k}\left(i\right)$ is the *i*’th element in the vector ${\underline{e}}_{k}$.

Plugging (18) into (17) and rearranging, we get,

(19) $$\begin{array}{c} {\underline{g}}^{k+1}=\arg\min_{\underline{g}}{∥{\underline{e}}_{k}-\tilde{E_{k}}\underline{g}∥}^{2} \end{array}$$

where $\tilde{E_{k}}$ is defined as,

(20) $$\begin{array}{cc} \; & \tilde{E_{k}}=\left(\begin{array}{cccc} e_{k}\left(N-1\right) & e_{k}\left(N-2\right) & \ldots & e_{k}\left(N-p\right)\\ e_{k}\left(N-2\right) & e_{k}\left(N-3\right) & \ldots & e_{k}\left(N-\left(p+1\right)\right)\\ \ldots & \ldots & \ldots & \ldots \end{array}\right) \end{array}$$

We notice that (19) can be solved using LS and the solution is,

(21) $${\underline{g}}^{k+1}={\left({\tilde{E_{k}}}^{†}\tilde{E_{k}}+\lambda_{g}I_{M}\right)}^{-1}{\tilde{E_{k}}}^{†}{\underline{e}}_{k}$$

where $\lambda_{g}$ is a regularization factor added to increase robustness of the estimation.

We notice that (21) is equivalent to the Yule–Walker solution for the AR parameters estimation.

### 3.6. Convergence of Alternating Minimization

In this section, the convergence of the proposed algorithm is analyzed. Unfortunately, there is no guarantee that the alternating minimization algorithm for the problem defined in (13) provides the optimal set of filters. However, using the results from [29], it is possible to prove that the algorithm converges to a stationary point and, thus, a real time implementation of the system is useful.

Consider the following optimization problem for x∈X,y∈Y:

$$\min_{x,y}f\left(x,y\right)$$

In [29], the following theorem is provided for block coordinate descend algorithms:

**Theorem** **1.** (Grippo and Sciandrone (2000)). *Let f be continuously differentiable and X,Y the closed and convex sets. Assuming both sub-problems have solutions and that the sequence $\left\{\left(x^{k},y^{k}\right)\right\}$ has limit points. Then, every limit point is stationary.*

The alternating minimization algorithm proposed in this paper is equivalent to a block coordinate descend of (13) when the optimization is done on two blocks: $\underline{g}$ and $\underline{h}$. Each least squares optimization is performed with regularization. This is equivalent to least squares with a Euclidean norm constraint on $\underline{h}$ and $\underline{g}$. Therefore, the possible filter vectors belong to closed sets.

**Lemma** **1.** *The set *Ω* defined as the set containing all the vectors $\underline{h}\in R^{n}$ such that ${∥\underline{h}∥}^{2}\leq \lambda$ is convex and closed.*

**Proof.**  The claim that Ω is closed is trivial. For two vectors, ${\underline{h}}_{1}\in \Omega$ and ${\underline{h}}_{2}\in \Omega$ with 0≤α≤1

(22) $$\begin{array}{cc} \; & {∥\alpha {\underline{h}}_{1}+\left(1-\alpha \right){\underline{h}}_{2}∥}^{2}\\ & ={∥\alpha {\underline{h}}_{1}∥}^{2}+{∥\left(1-\alpha \right){\underline{h}}_{1}∥}^{2}+2\alpha \left(1-\alpha \right){\underline{h}}_{1}^{T}{\underline{h}}_{2} \end{array}$$

Using the fact that ${\underline{h}}_{1},{\underline{h}}_{2}\in \Omega$ and the Cauchy–Schwartz inequality,

$${∥\alpha {\underline{h}}_{1}+\left(1-\alpha \right){\underline{h}}_{2}∥}^{2}\leq \alpha^{2}\lambda +{\left(1-\alpha \right)}^{2}\lambda +2\alpha \left(1-\alpha \right)\lambda$$

This means that every convex combination of two vectors in the set Ω satisfies:

$${∥\alpha {\underline{h}}_{1}+\left(1-\alpha \right){\underline{h}}_{2}∥}^{2}\leq \lambda$$

□

Therefore, the algorithm satisfies the conditions of Theorem 1 and the algorithm converges to a stationary point.

## 4. Low-Complexity Implementation Using RLS Submodules

**Figure 3:**
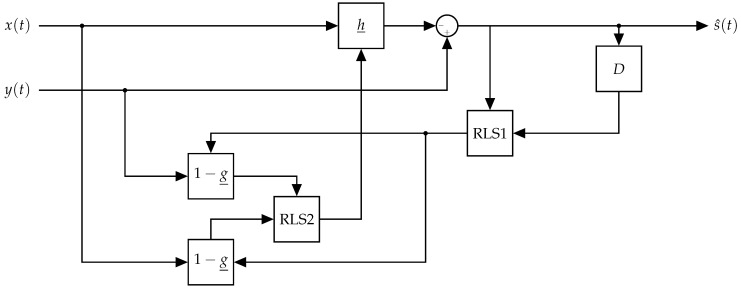
Joint Whitening RLS (JWRLS) algorithm flow diagram.

Algorithm 1 works on a batch of samples, which is not suitable for real time systems. LS problems can be converted to adaptive recursive least squares (RLS), providing a sequential, real time solution. The proposed algorithm, which we denote as Joint Whitening RLS (JWRLS), is detailed in Figure 3, and can be summarized as follows: the DL and UL signals go through a whitening filter $1-\underline{g}$ and are then fed to an RLS module that produces an estimate of the self-interference filter. In parallel, the reference is convolved with the self-interference filter estimate and subtracted from the UL, which produces an estimate of the UL without interference. This output is delayed by one sample and sent to another RLS module, which estimates the UL covariance, which is basically the whitening filter.

The RLS has many implementations. One notable implementation in low-complexity is the RLS-DCD [24]. RLS-DCD algorithm is a low-complexity approximation of RLS. This algorithm has a linear (with the filter’s length) number of real multipliers and, thus, it is much simpler to implement than conventional RLS. In [30], there is also an FPGA implementation of this algorithm, which shows its real world value and applicability. The JWRLS-DCD will denote the JWRLS implementation using DCD.

The classical RLS adaptive algorithm uses an initial regularization to stabilize the solution to the RLS problem [31]. Because the initial regularization decays exponentially in time, we may have to add additional diagonal loading to maintain robustness [32]. However, such extra diagonal loading increases the complexity to $O(N^{3})$ [31], which makes the RLS algorithm impractical [32]. In [33], the authors propose modifying the adaptive RLS-DCD algorithm introduced in [24] to incorporate the diagonal loading factor.

## 5. Performance Analysis

In this section, the proposed algorithm is compared to other state-of-the-art self-interference cancellation algorithms. The comparison is for computational complexity and interference cancellation quality. It will be shown that the proposed algorithm provides the best trade-off between computational complexity and interference cancellation.

### 5.1. Computational Complexity

The computational complexity is measured in terms of the number of real multipliers needed for the implementation of each algorithm. In Table 1, the parametric expression for the computational complexity for several state-of-the-art algorithms is detailed. All of these algorithms will be compared in terms of interference cancellation in Table 2.

Since the proposed algorithm (JWRLS DCD) is composed of two DCD-RLS modules, its complexity is linear with the filter size. The only algorithms that scale the same are based on LMS, which perform poorly when the spectrum of the UL is not white.

### 5.2. Simulation Results

In this section, the proposed algorithm’s performance is analyzed using a simulation. DL and UL signals were generated by colored Gaussian processes with variable power levels and bandwidths. In order to simulate the PA’s response, non linearity was introduced to the DL signal using a Hammerstein–Wiener model [35]. The DL signal was filtered with a measured lab channel response to simulate the RF leakage filter. Finally, the self-interference was added to the UL, which was then inputted to our algorithm. Note that JWRLS-DCD used 24 taps for both filters $\underline{g}$ and $\underline{h}$. All other algorithms used 24 taps for their filter estimation.

In Table 2, the JWRLS-DCD algorithm is compared with state-of -the-art algorithms from Table 1, in terms of UL SNR performance. This SNR is computed by first taking the spectral difference between the clean UL (without DL leakage) and the estimated UL. This difference is the residual noise on the UL signal. Next, the SNR is computed by taking the UL power and dividing it with the power of the residual estimation noise. The SNR values represent the mean SNR across the BW and have been rounded to the nearest 5 dB.

Three different scenarios, which represent typical multi access scenarios, were examined. The resulting absolute rejection in dB across the frequency domain is plotted for each scenario. In each figure, we denote the TX signal (DL) as REF, the leakage DL in RX as interference, the thermal noise floor as noise, and the DL rejection per algorithm as the residual noise per each mitigation algorithm. The hope is to have the residual noise of a specific mitigation algorithm below the thermal noise floor.

**Figure 4:**
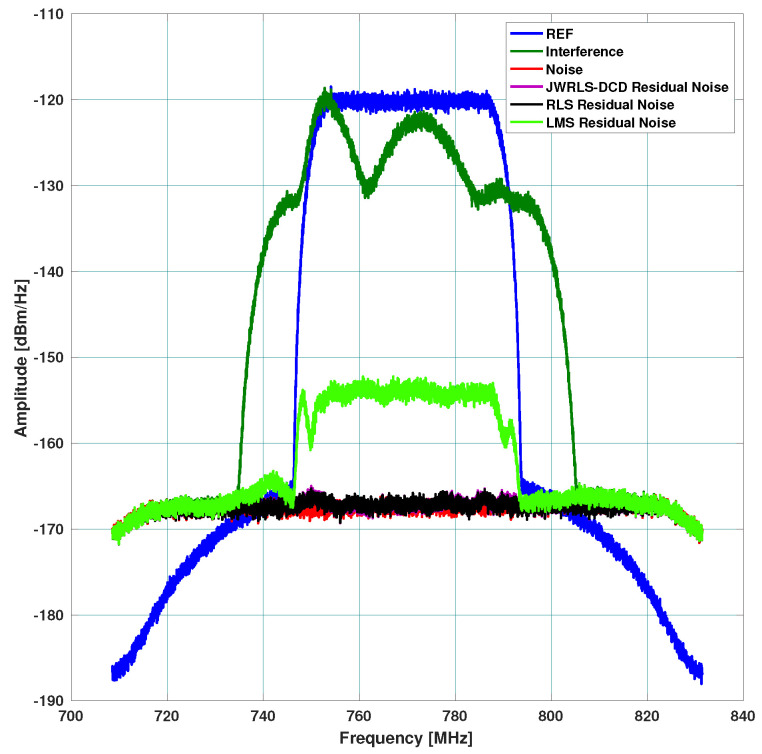
Rejection performance for wideband UL-RLS and LMS.

**Figure 5:**
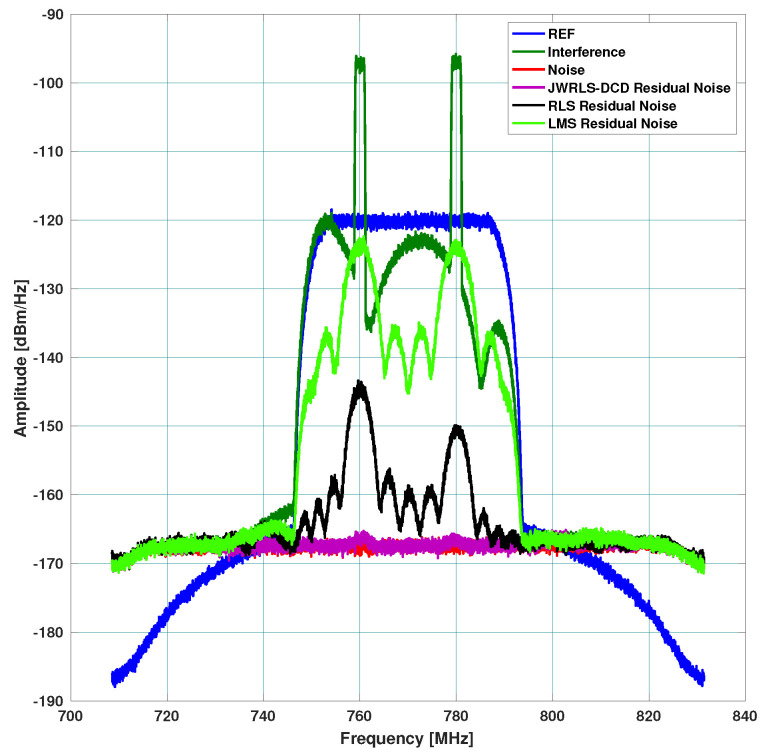
Rejection performance for two narrow band UL-LMS and RLS.

**Figure 6:**
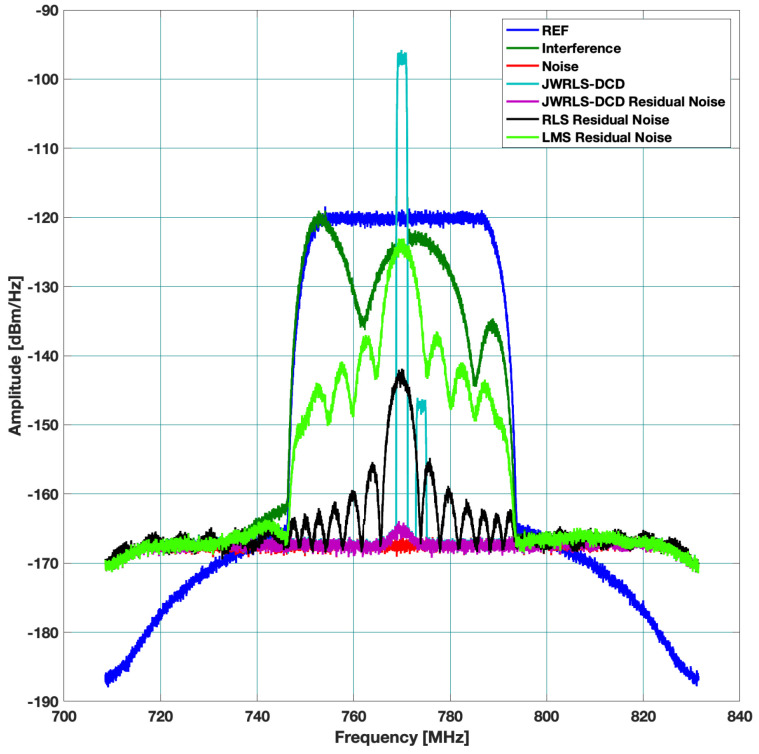
Rejection performance for near far narrow band UL-LMS and RLS.

**Figure 7:**
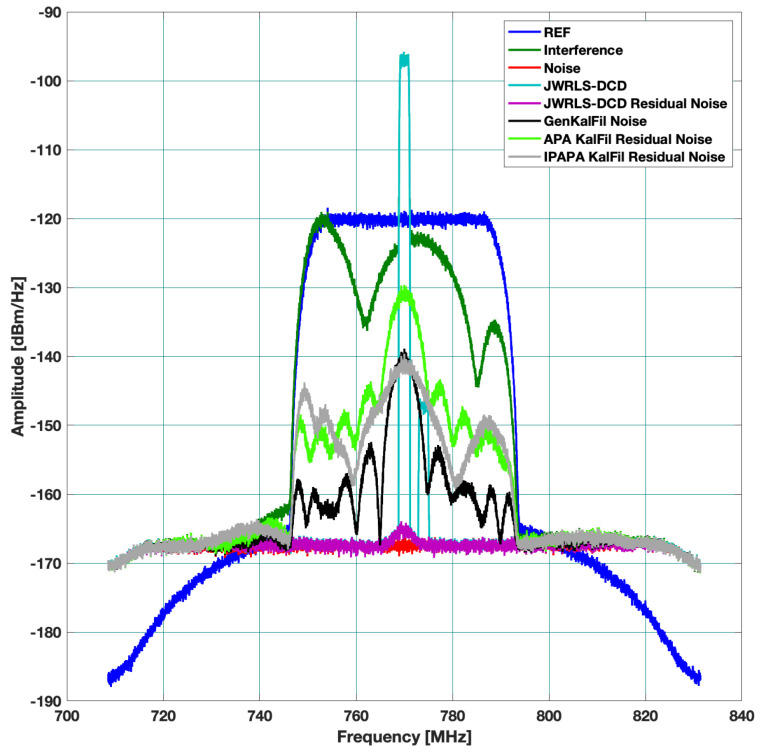
Rejection performance for near far narrow band UL-TDAKF.

**Figure 8:**
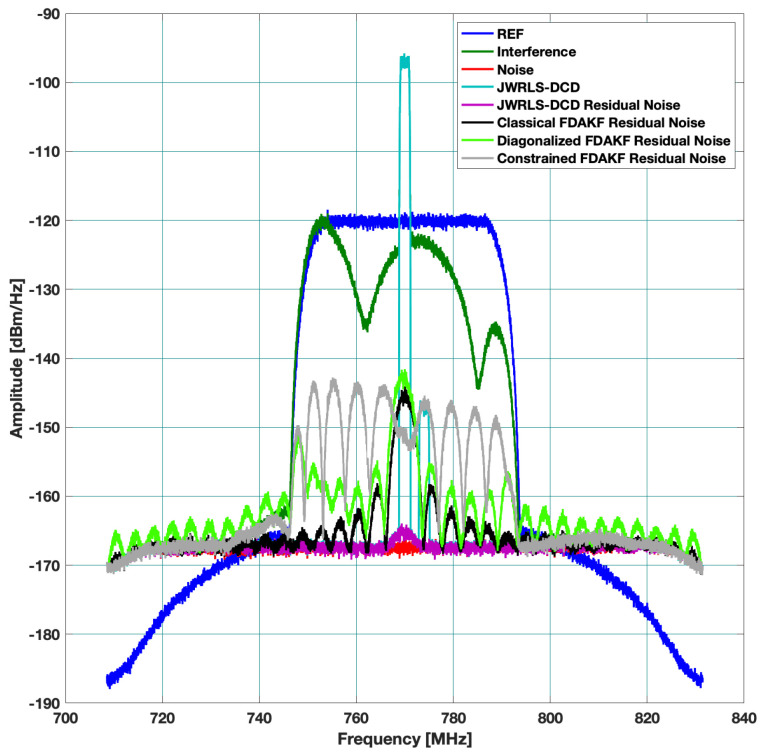
Rejection performance for near far narrow band UL-FDAKF.

1. Scenario A: includes a 40 MHz UL and higher power 30 MHz DL. The DL undergoes the leakage filter and a rippled DL frequency response is present at the RX input. The performance of the interference cancellation of JWRLS-DCD is comparable to the RLS in this case since the UL is essentially white in the frequency band of interest. Note that the computational complexity of JWRLS-DCD is the same order as RLS. It is also shown that LMS performs very poorly in this case compared to RLS and JWRLS-DCD. The performance comparison with RLS and LMS is shown in Figure 4.
2. Scenario B: includes two 2 MHz UL signals at a 20 MHz carrier difference and 40 MHz DL with different power levels. This scenario exemplifies the issue of a non-white UL signal, which can deteriorate the LS interference cancellation. Indeed the performance of the interference cancellation of JWRLS-DCD is much better than all of the other algorithms, some areas are even 10 dB better. The performance comparison with RLS and LMS is shown in Figure 5.
3. Scenario C: comprises of two narrow band UL signals (2 MHz each) at 4 MHz carrier spacing with a significant power difference. This scenario simulates a “near- far” issue where a nearby user is masking a far user and limits the service range of the access point. In Figure 6, the performance of JWRLS-DCD is shown and we can see that the weak user is recovered along with the strong user and essentially the residual is at the noise floor. In this case, we also added the JWRLS estimated UL to show the weak and strong users and exemplify the fact that the weak user is masked by the residual noise of the other algorithms. It is clearly shown that JWRLS-DCD achieves the best performance in this demanding scenario. Since this is an interesting and demanding scenario, we also included the UL SNR for TDAKF and FDAKF-based algorithms in Figure 7 and Figure 8. Clearly, JWRLS-DCD is the only algorithm providing sufficient rejection for the weak user recovery.

In conclusion, in all three scenarios, JWRLS-DCD achieved the best performance while maintaining low computational complexity. It is worth noting that in scenario C (near far problem), JWRLS-DCD was the only algorithm to provide sufficient SNR for the reception of the weak user, enabling to extend the operational range of the base station!

### 5.3. Performance for Time Varying Channels

In this section, the performance of the proposed scheme is analyzed for time varying channels, in particular, channels with Doppler spread. In practical implementations of a full duplex system, the leakage filter may vary slowly over time due to movement of close range users or the movement of the base station/access point. This movement can alter different multi-path components and slowly change the channel response of the leakage. This slow change of the channel response is modeled using a Doppler power spectrum. In order to adapt to the varying channel, the proposed self-interference rejection scheme includes a forgetting factor, which helps track the changing leakage filter.

In this simulation, Jakes’ model [25] was chosen to model a Doppler power spectrum. The simulation consists of first generating *N* independent stochastic processes $a_{i}\left(t\right)$ for different multi paths in the leakage channel. Each process, $a_{i}\left(t\right)$, is generated by passing a complex Gaussian sequence through a U-shaped filter sampled at 1 KHz rate. The output of this filter is then multiplied by the average power of the path to adjust the variance and interpolated to the system’s sampling rate.

The self-interference, y(t) can be written as:

$$y\left(t\right)=\sum_{i=0}^{N}a_{i}\left(t\right)s\left(t-\tau_{i}\right)$$

where s(t) and $\tau_{i}$ are the DL signal and delay of multi path ray *i*, respectively.

**Figure 9:**
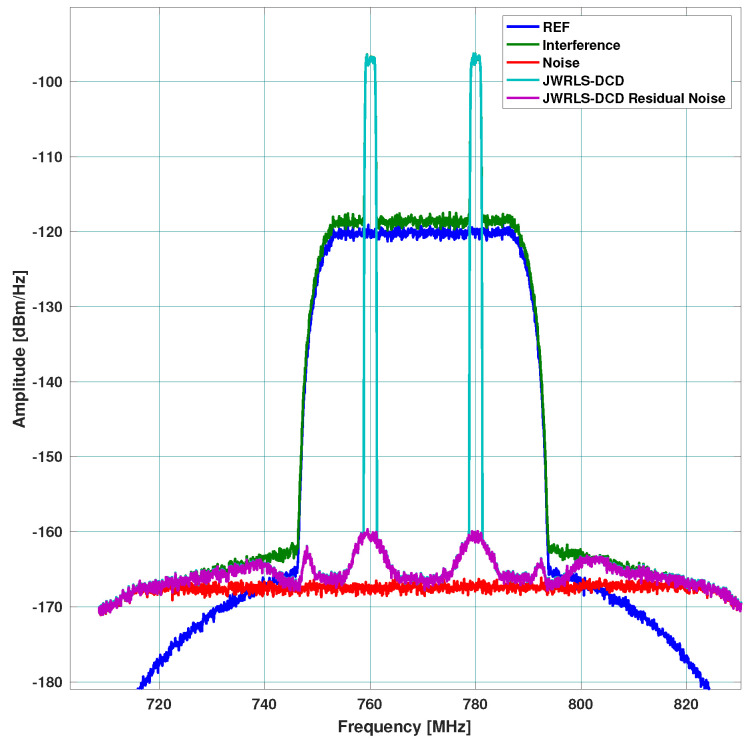
Rejection performance for two narrow band UL signal scenarios with a 2-tap leakage filter and Doppler of 120 Hz.

**Figure 10:**
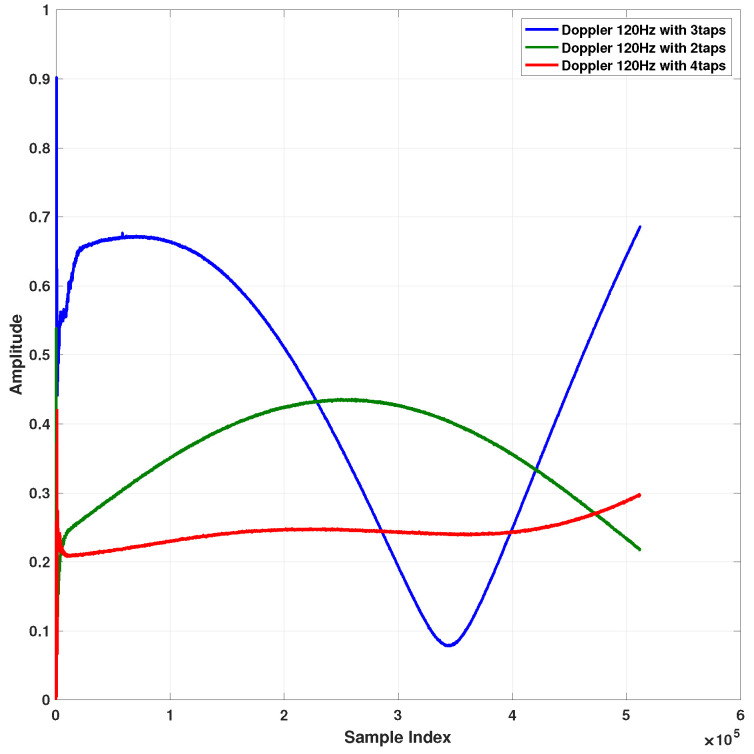
Estimated first tap of leakage filter for different leakage filters.

A maximal Doppler frequency of 120 Hz was chosen and delay profiles with 2, 3, and 4 taps with the parameters detailed in Table 3 were examined. In Figure 9, the residual noise after the removal of the leakage is presented and the performance is with negligible loss compared to the time-invariant channel setting. In Figure 10, the evolution of the first tap in the leakage filter is plotted for 2, 3, and 4 tap channels. The tracking shows that the first tap changes over time and considering the fact that the residual interference is very close to the noise floor (as for a fixed leakage as shown in Figure 9), we can conclude that the tracking is good.

## 6. Conclusions

In this paper, a novel algorithm for mitigating self-interference in full duplex communication systems was presented. This algorithm is capable of mitigating self-interference in multiple access systems, where multiple users are simultaneously received and transmitted at overlapping arbitrary bandwidths and powers. The main innovation was the stochastic modeling of the UL signal as an AR process and the joint estimation of the leakage and AR parameters. Furthermore, a low-complexity implementation was proposed using the RLS-DCD scheme, which resulted in an algorithm whose computational complexity scales linearly with the filter size.

Finally, an extensive comparison to a large number of state-of-the-art algorithms for self-interference mitigation was carried out, and it demonstrated the superiority of the proposed algorithm.

## Figures and Tables

**Table 1:** Computational complexity of algorithms.

Algorithm	Real Multiplications per Sample	Comments
JWRLS DCD	60L	*L* is the filter size
VSS-NLMS1 [12]	20L+34	
VSS-NLMS2 [13]	8L+22	
VSS-NLMS3 [14]	20L+41	
VSS-APA [15]	$4p^{3}+8p^{2}+8pL+8p+12$	*p* is the projection order
MDF [34]	$8K+\left(4K+6\right)\log_{2}N$	*K* sub-filters, *N* is the sub-filter length
IPMDF [34]	$10K+\left(4K+6\right)\log_{2}N$	
EMDF [17,18]	$48K-8K/N+18-12/N+4\left(2K+3\right)\log_{2}2N$	
General KF [21]	$4L^{3}+8L^{2}p+8Lp^{2}+8Lp+4p^{3}$	
APA KF [21]	$8Lp+12p^{2}+4p^{3}$	Complexity without noise statistic calculation.
IPAPA KF [21]	$4Lp^{2}+12Lp+4L+4p^{3}+4p^{2}$	
Classical FDAKF [22]	$24M^{3}+12M^{2}+1.5M\log_{2}M-3\frac{3}{4}M$	*M* is the length of FFT.
Diagonalized FDAKF [22]	$1.5M\log_{2}M+20M^{2}+6\frac{1}{4}M$	
Constrained FDAKF [22]	$2.5M\log_{2}M+20M^{2}-3\frac{3}{4}M$	

**Table 2:** UL SNR Comparison.

Algorithm	Scenario A UL SNR [dB]	Scenario B UL SNR [dB]	Scenario C UL SNR [dB]
JWRLS DCD	50	70	20
RLS & LS	50	50	0
LMS	35	35	−15
VSS-NLMS1 [12]	35	40	−10
VSS-NLMS2 [13]	35	40	−10
VSS-NLMS3 [14]	35	40	−10
VSS-APA [15]	20	40	−10
EMDF [17,18]	35	40	0
General KF [21]	45	60	5
APA KF [21]	25	40	−10
IPAPA KF [21]	30	50	0
Classical FDAKF [22]	50	65	10
Diagonalized FDAKF [22]	45	60	10
Constrained FDAKF [22]	25	50	0

**Table 3:** Parameters for Jakes’ model.

Number of Taps	Delays [ns]	Amplitudes
2	0,2	1,0.3
3	0,3,10	1,0.3,0.1
4	0,3,7,11	1,0.5,0.3,0.1
